# Supplementary material for: Multi‐dataset identification of innovative feature genes and molecular mechanisms in keratoconus
Source: J Cell Mol Med. 2024 Sep 19;28(18):e70079. doi: 10.1111/jcmm.70079 (PMC11412914; doi:10.1111/jcmm.70079)
Supplement: Supplementary file 2 — Material S2. [file JCMM-28-e70079-s002.zip › 7.GSEA analysis/2.ATOH7/gsea_report_for_l_1678852790847.html]

Report for l 1678852790847 [GSEA]

| GS  follow link to MSigDB | GS DETAILS | SIZE | ES | NES | NOM p-val | FDR q-val | FWER p-val | RANK AT MAX | LEADING EDGE || 1 | KEGG\_PENTOSE\_AND\_GLUCURONATE\_INTERCONVERSIONS | Details ... | 20 | -0.49 | -1.28 | 0.144 | 1.000 | 0.953 | 3779 | tags=25%, list=11%, signal=28% |
| 2 | KEGG\_PORPHYRIN\_AND\_CHLOROPHYLL\_METABOLISM | Details ... | 32 | -0.35 | -1.13 | 0.322 | 1.000 | 0.994 | 5930 | tags=19%, list=17%, signal=23% |
| 3 | KEGG\_STEROID\_BIOSYNTHESIS | Details ... | 17 | -0.36 | -1.11 | 0.333 | 1.000 | 0.995 | 10973 | tags=59%, list=32%, signal=86% |
| 4 | KEGG\_PROTEASOME | Details ... | 43 | -0.31 | -1.11 | 0.263 | 1.000 | 0.996 | 23961 | tags=100%, list=69%, signal=322% |
| 5 | KEGG\_PHENYLALANINE\_METABOLISM | Details ... | 17 | -0.37 | -1.02 | 0.439 | 1.000 | 1.000 | 2394 | tags=18%, list=7%, signal=19% |
| 6 | KEGG\_HOMOLOGOUS\_RECOMBINATION | Details ... | 26 | -0.30 | -1.02 | 0.433 | 1.000 | 1.000 | 2100 | tags=8%, list=6%, signal=8% |
| 7 | KEGG\_ASCORBATE\_AND\_ALDARATE\_METABOLISM | Details ... | 17 | -0.36 | -0.99 | 0.445 | 1.000 | 1.000 | 3779 | tags=24%, list=11%, signal=26% |
| 8 | KEGG\_GLIOMA | Details ... | 65 | -0.23 | -0.98 | 0.472 | 1.000 | 1.000 | 1949 | tags=8%, list=6%, signal=8% |
| 9 | KEGG\_RIBOSOME | Details ... | 85 | -0.25 | -0.93 | 0.504 | 1.000 | 1.000 | 26040 | tags=100%, list=75%, signal=399% |
| 10 | KEGG\_PROTEIN\_EXPORT | Details ... | 22 | -0.29 | -0.93 | 0.577 | 1.000 | 1.000 | 24838 | tags=100%, list=72%, signal=351% |
| 11 | KEGG\_GLYCOSAMINOGLYCAN\_BIOSYNTHESIS\_HEPARAN\_SULFATE | Details ... | 26 | -0.27 | -0.89 | 0.608 | 1.000 | 1.000 | 5827 | tags=19%, list=17%, signal=23% |
| 12 | KEGG\_GLYOXYLATE\_AND\_DICARBOXYLATE\_METABOLISM | Details ... | 16 | -0.30 | -0.86 | 0.612 | 1.000 | 1.000 | 3803 | tags=13%, list=11%, signal=14% |
| 13 | KEGG\_TERPENOID\_BACKBONE\_BIOSYNTHESIS | Details ... | 15 | -0.32 | -0.83 | 0.634 | 1.000 | 1.000 | 11534 | tags=60%, list=33%, signal=90% |
| 14 | KEGG\_AMINOACYL\_TRNA\_BIOSYNTHESIS | Details ... | 22 | -0.26 | -0.79 | 0.743 | 1.000 | 1.000 | 25846 | tags=100%, list=74%, signal=391% |
| 15 | KEGG\_OOCYTE\_MEIOSIS | Details ... | 111 | -0.15 | -0.75 | 0.816 | 1.000 | 1.000 | 8022 | tags=15%, list=23%, signal=20% |
| 16 | KEGG\_GLYCOSPHINGOLIPID\_BIOSYNTHESIS\_GANGLIO\_SERIES | Details ... | 15 | -0.26 | -0.73 | 0.742 | 1.000 | 1.000 | 12968 | tags=60%, list=37%, signal=96% |
| 17 | KEGG\_BETA\_ALANINE\_METABOLISM | Details ... | 22 | -0.25 | -0.73 | 0.861 | 1.000 | 1.000 | 3722 | tags=14%, list=11%, signal=15% |
| 18 | KEGG\_SPLICEOSOME | Details ... | 125 | -0.16 | -0.71 | 0.877 | 1.000 | 1.000 | 24807 | tags=96%, list=71%, signal=335% |
| 19 | KEGG\_PANTOTHENATE\_AND\_COA\_BIOSYNTHESIS | Details ... | 16 | -0.26 | -0.68 | 0.873 | 1.000 | 1.000 | 3095 | tags=13%, list=9%, signal=14% |
| 20 | KEGG\_ECM\_RECEPTOR\_INTERACTION | Details ... | 84 | -0.20 | -0.65 | 0.925 | 1.000 | 1.000 | 9688 | tags=33%, list=28%, signal=46% |
| 21 | KEGG\_GLYCOSYLPHOSPHATIDYLINOSITOL\_GPI\_ANCHOR\_BIOSYNTHESIS | Details ... | 25 | -0.20 | -0.63 | 0.868 | 1.000 | 1.000 | 27826 | tags=100%, list=80%, signal=503% |
| 22 | KEGG\_ABC\_TRANSPORTERS | Details ... | 44 | -0.19 | -0.57 | 1.000 | 1.000 | 1.000 | 8504 | tags=25%, list=24%, signal=33% |
| 23 | KEGG\_BIOSYNTHESIS\_OF\_UNSATURATED\_FATTY\_ACIDS | Details ... | 19 | -0.17 | -0.56 | 0.975 | 1.000 | 1.000 | 12936 | tags=53%, list=37%, signal=84% |
| 24 | KEGG\_NOTCH\_SIGNALING\_PATHWAY | Details ... | 47 | -0.14 | -0.54 | 0.960 | 0.977 | 1.000 | 7875 | tags=15%, list=23%, signal=19% |
Table: Gene sets enriched in phenotype **l (8 samples) **[plain text format]****
